# Adenoid cystic carcinoma of cervix in younger women: report of two new cases

**DOI:** 10.11604/pamj.2014.19.99.5241

**Published:** 2014-09-29

**Authors:** Mohamed Sinaa, Mohamed Oukabli, Abderahmane Albouzidi

**Affiliations:** 1 Department of Pathology, Military Hospital Moulay Ismail, Meknes, Morocco; 2 Department of Pathology, Military Hospital Mohamed V, Rabat, Morocco

**Keywords:** Adenoid cystic carcinoma, cervix, radiotherapy

## Abstract

Adenoid cystic carcinoma is a malignant epithelial neoplasm derived from the salivary glands. Primary adenoid cystic carcinoma of the cervix is extremely rare, accounting for less than 1% of all cervical carcinomas. Its origin is debatable. It generally presents in elderly age group, however only twenty three cases have been reported in women less than age 45 years old. In this paper we report two new cases of primary adenoid cystic carcinoma in younger women and include the cytopathology and histopathology findings. A 36, 41 year-old women were admitted with signs and symptoms suggestive of a cervical cancer. Speculum examination showed a firm mass in the uterine cervix. The diagnosis of adenoid cystic carcinoma was confirmed by the histopathology examination of the biopsy specimen in the two patients, which characteristically revealed the presence of a cribriform pattern and cylindromatous structures of the tumor cells, adhered around hyaline stoma. They were managed successfully by concurrent radiotherapy. Adenoid cystic carcinoma of the cervix is a rare disease. It usually occurs in an elderly age group. We report these two cases because of its rarity in young patients, with description of illustrative pathology and discussion on the histological diagnosis.

## Introduction

Adenoid cystic carcinoma (ACC) is a malignant epithelial neoplasm derived from the salivary glands and can occur in a variety of other sites. It is characterized by slow growth and high rate of local recurrence. Primary ACC of the cervix is extremely rare, accounting for less than 1% of all cervical carcinomas [1]. It is usually seen in postmenopausal, often multigravid black women, but rarely can develop in patients under 45 [2]. We report two cases of primary ACC of the cervix in younger patient, and we discuss briefly the clinical, pathological and therapeutic features of the disease.

## Patient and observation

**Case 1**: A 36 year-old female presented with vaginal bleeding for six months. Per vaginal and per speculum examination revealed firm mass in the uterine cervix. The radiologic investigations revealed an mass of 2 cm in region of cervix. The patient was staged IB1 according to classification of International Federation of Gynecology and Obstetrics (FIGO) for carcinoma cervix.

**Case 2**: A 41 year-old female, presented with the same symptoms with lumbar pain. General physical examination was normal. Pelvic computed tomography (CT) scan showed a mass of 4 cm over the cervical region. The patient was staged IIA of FIGO. Biopsy of the cervical lesion was done and sent for histopathological examination for the two patients.

**Figure 1:**
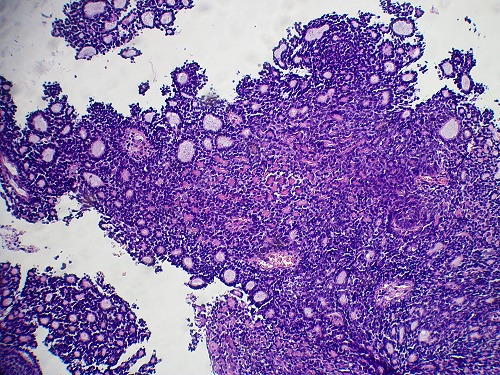
The biopsy specimen of case 1: Proliferation composed of trabecular structure. Cylindromatous structures were present. The cribriform pattern was barely seen (Hematoxylin and eosin (HE) x 100)

**Figure 2:**
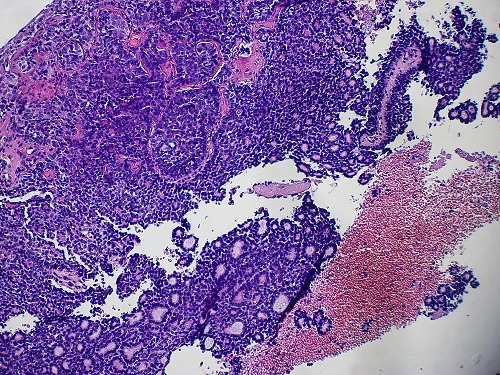
ACC: combining tubular and solid features (case 2) (HE x 100)

**Figure 3:**
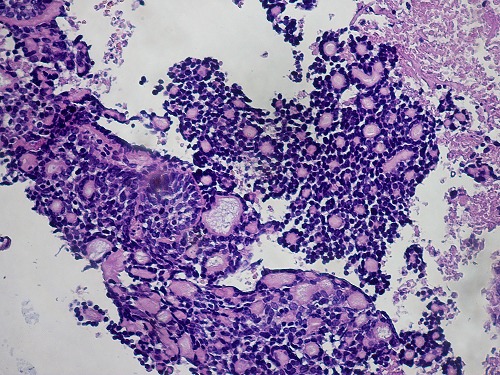
ACC: The tumor cells were small, basaloid, uniform, composed of dense basophilic nuclei with inconspicuous nucleoli (case 1,2) (HEx 200)

**Figure 4:**
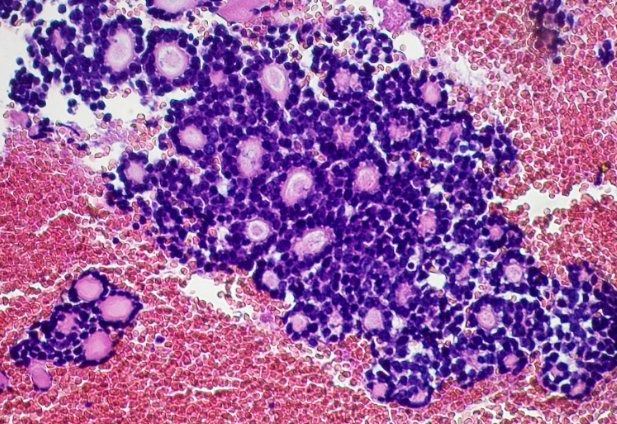
ACC: The tumor cells were small, basaloid, uniform, composed of dense basophilic nuclei with inconspicuous nucleoli (case 2) (HE x 200)

The two neoplasms were histologically similar. The proliferation disposed in cribriform nests and cords. The hyaline stroma forming cyst like spaces within cell nests revealed a classic cylindromatous structures (Figure 1, Figure 2). The tumor cells were small, uniform, composed of dense basophilic nuclei with inconspicuous nucleoli. Mitotic figures were rarely found (Figure 3, Figure 4). A diagnosis of adenoid cystic carcinoma was made. The patients were managed successfully by radiotherapy followed by hysterectomy. The postoperative period was uneventful and the patients are on regular follow-up for last nine months.

## Discussion

Primary ACC of the cervix accounts for less than 1% of all cervical carcinomas [1-3]. The origin of this disease is still unknown. Although, Human papillomavirus (HPV) infection is believed to be a necessary cause of cervical cancer, its role in the pathogenesis of ACC is not well defined [3, 4]. The most accepted view regarding its origin in the cervix is from “reserve cells” of endocervix [4, 5]. There are some controversies concerning the epidemiological features of this tumor. ACC of cervix was considered as the disease of the postmenopausal women, it's rarely occurs in younger patients. Only twenty three cases have been reported in women less than age 45 years-old [3, 4]. Although, some authors suggested the association between ACC, high parity and black race [5-7]. Clinicaly, it presents as a non friable mass on speculum examination, in contrast to the friable growth usually seen in squamous cell carcinoma of cervix. Most of the cases present with vaginal bleeding as chief complaint. The most majority of patients were diagnosed at early stages [6, 7]. Microscopically, the morphologic appearance is similar to that of homonymous tumors of salivary, the ACC a pattern described as cribriform: nests and columns of cells of rather bland appearance are arranged concentrically around glandlike spaces (‘pseudocysts’) filled. Most of these are not true glandular spaces; they represent instead extracellular cavities containing reduplicated basal lamina material and mucin produced by the tumor cells [5]. Small true glandular lumina are also formed. Indeed, identification of both pseudocysts and true glandular lumina is required to make a diagnosis of adenoid cystic carcinoma. This tumor has a remarkable tendency for invasion of perineurial spaces [5]. It's seems to be more aggressive than squamous cell carcinoma of the cervix, with higher tendency to local and metastatic recurrence even if diagnosed in their earliest stages. Five years and ten years survival were 37% and 40% respectively. These tumours spread most frequently to the lung, lymph nodes, abdominal cavity and brain. The prognostic factors identified were: large tumour diameter, and vascular emboli [6-9] Because of the rarity of the disease and the absence of prospective studies, no standard treatment has yet been proposed. Most patients were treated as squamous cell carcinoma. Surgery seems to be the treatment of choice in combination with adjuvant radiotherapy and/or chemotherapy, based on the clinical stage and presence of metastasis. Actually, ACC of the cervix is considered a radiosensitive tumor [10].

## Conclusion

ACC is a rare variant of adenocarcinomas of the uterine cervix. As only some case reports are available in the literature in the younger women, further studies and metaanalysis of the reported cases is required to assess high risk factors and to design a comprehensive treatment strategy.
